# Is Inhibition of Return Modulated by Involuntary Orienting of Spatial Attention: An ERP Study

**DOI:** 10.3389/fpsyg.2017.00113

**Published:** 2017-01-31

**Authors:** Fada Pan, Xiaogang Wu, Li Zhang

**Affiliations:** The Department of Applied Psychology, School of Education Science, Nantong UniversityNantong, China

**Keywords:** inhibition of return, reorienting hypothesis, peripheral cue, central cue, event-related potentials

## Abstract

Inhibition of return (IOR) is a mechanism that indicates individuals’ faster responses or higher accuracy to targets appearing in the novel location relative to the cued location. According to the “reorienting hypothesis,” disengagement from the cued location is necessary for the generation of IOR. However, more and more studies have questioned this theory because of dissociation between voluntary or involuntary spatial orienting and the IOR effect. To further explore the “reorienting hypothesis” of IOR, the present experiment employed an atypical cue-target paradigm which combined a spatially non-predictive peripheral cue that was presumed to trigger IOR with a spatially non-predictive central cue that was used to reflexively trigger a shift of attention. The results showed that a significant IOR effect did not interact with automatic spatial orienting as measured in mean RTs and accuracy as well as the Nd component. These findings suggested that the IOR effect triggered by peripheral cue was independent of automatic orienting generated by a central cue. Therefore, the present study provided evidence from location task and neural aspects, which again challenged the “reorienting hypothesis” of IOR.

## Introduction

Attention orienting can be induced voluntarily or involuntarily by a variety of stimuli. Traditional literature has described two different mechanisms: endogenous orienting and exogenous orienting, which represented inner goals and external demands respectively (Jonides, 1981; Posner and Cohen, 1984; Klein, 2000; Chica et al., 2011). The common operation is that researchers present a predictive central cue (e.g., an eye-gaze or arrow that points to the left or right) at fixation to study endogenous orienting, and present a non-predictive peripheral cue in the left or right to study exogenous orienting (Ruz and Castillo, 2002). Orienting of attention in both types of cues involves three processes of engagement, disengagement and re-orienting, and the cue-target paradigm has been widely used to investigate these processes (Posner, 1980).

Typically, a non-informative peripheral cue can produce facilitatory effect or inhibitory effect based on different stimulus onset asynchronies (SOA). If the cue-target SOA is less than 300 ms, a facilitatory effect can be observed, showing faster reaction times (RTs) at the cued location (cue and target appear on the same side) than those at the uncued location (cue and target appear on different sides). On the contrary, if the cue-target SOA is longer than 300 ms, early facilitatory effect will be replaced by an inhibitory effect. That is the well-known inhibition of return (IOR) (Posner et al., 1985), which refers to the faster response at the uncued location than that at the cued location (Posner and Cohen, 1984). However, when the central cue is presented at fixation, a long-lasting facilitatory effect can be observed even at a longer SOA (e.g., 1000 ms): individuals generally respond faster to targets appearing at a location indicated by the central cue than those not indicated by a central cue (Funes et al., 2007). Psychologists explain that IOR is a mechanism reflecting adaptability and flexibility of the human cognitive process by encouraging attention shift to a novel location and inhibiting attention from re-orienting back to an inspected location (MacInnes and Klein, 2003; Ivanoff and Taylor, 2006). According to this “re-orienting hypothesis,” attentional disengagement is essential for generating the IOR effect: when the attention is attracted by a peripheral cue, the cued location first produces a facilitatory effect; then, after a few 100 ms, the attention is disengaged from the cued location and an inhibitory effect starts to prevent the attention from returning to that cued location.

Even though the explanation of the “re-orienting hypothesis” of IOR was widely accepted by many researchers in the attention field, many studies have focused on the voluntary attention and they found a robust IOR effect when attentional disengagement did not take place. Researchers used predictive peripheral cues to guarantee that indicated and non-indicated trials can be either cued or uncued in both detection and discrimination tasks (Chica et al., 2006). The results showed that a typical IOR effect was consistently found at the indicated location. Berger et al. (2005) used a paradigm with a predictive central cue and a non-predictive peripheral cue to investigate the relation between IOR and voluntary orienting (a predictive central cue) of visual attention. In the experiment, the validity of an endogenously predictive central cue was 80% (i.e., the target appeared at the indicated location with 80% probability). The IOR effect was observed not only at the indicated location but also at the non-indicated location. Moreover, there is a growing body of work showing that the disengaging of attention seems to be unnecessary or insufficient (Danziger and Kingstone, 1999; Chica and Lupiáñez, 2004, 2009; Lupiáñez, 2010, chapter 2).

Recently, Martín-Arévalo et al. (2013) combined both non-predictive peripheral cue and non-predictive central cue to demonstrate the separation from the IOR effect and involuntary orienting of spatial attention. In their experiments, the peripheral cue was manipulated by presenting a salient cue in one of four positions (up, down, left, and right), and involuntary orienting was operated by the central cue pointing to one of the four potential target locations. The results were clear: no matter how the central cue was operated, the interactions between peripheral cue and central cue didn’t reach a significant level (Martín-Arévalo et al., 2013). The set of implied cues (e.g., gazing face, gazing eye or arrow) had a significant facilitatory effect indicating reflexive orienting to the target location (Friesen and Kingstone, 2003). Although centrally fixated directional stimuli like gaze cues and arrow cues have been demonstrated to the generation of reflexive orienting with long or short SOA (Friesen and Kingstone, 1998; Tipples, 2002), Green and Woldorff (2012) raised a spatial-conflict explanation when they used predictive arrow cues to find large RT cueing effects at very short cue target intervals (Green and Woldorff, 2012). The conflict between the cue and the target among invalid trials might be induced by the persistent cue. Then, Green et al. (2013) examined the time course of uninformative-arrow-cue effect and found that no significant cueing effect was observed for both short-duration conditions and significant cueing effect was observed at the two shortest SOA for the long-duration cues. The cueing effect was only found in the temporal overlap of the cue and target (long duration). They suggested that their results supported a spatial-incongruency explanation rather than automatic attention-orienting accounts. Gayzur et al. (2014) also examined the time course of uninformative-gaze-cues and found that short duration cues led to facilitation at short SOA but not long SOA and long duration cues induced the cueing effect at short and long SOA (interpreted as automatic orienting). Martín-Arévalo et al. (2013) manipulated the long duration arrow cue at the long SOA and they found RT was faster when target appeared at the indicated position, reflecting automatically attentional allocation of indicated location. It seemed that reflexive orienting was related with long duration in which central cues overlapped temporally with the target at the long SOA, when spatial-conflict was related with long duration at the short SOA. Regardless of different interpretations of the central cueing effect, IOR can occur when the attention was disengaged voluntarily or involuntarily from the attended location. Although there were some evidences which argued against the “re-orienting hypothesis” of IOR, further experiments using different tasks or paradigms are required to improve the validity of this disagreement.

To further investigate the modulation of the validity effect induced by the central arrow cue over the IOR effect, in the atypical paradigm of Martín-Arévalo et al. (2013), the present experiment used the same procedure. The purpose of the present study was to replicate previous results (Berger et al., 2005; Chica et al., 2006; Martín-Arévalo et al., 2013) and further explore the neural correlates of dissociation between the IOR effect and automatically attentional orienting. Firstly, we employed an atypical cue-target paradigm similar to the one used by Martín-Arévalo et al. (2013), in which the non-predictive peripheral cue was presented before the non-informative central cue. However, in the current experiment, we modified the procedure into a more common IOR paradigm (i.e., only the left and right locations). Additionally, we chose only the arrow cue to orient attention. Motoric and visual were two forms of IOR relating to activation and suppression of the oculomotor system, respectively (Taylor and Klein, 2000). Suppression of the oculomotor system often uses a localization task and a detection task, during which participants are required to keep their eyes on a fixation point. In the present experiment, a localization task was used instead of a detection task (Martín-Arévalo et al., 2013).

Our second aim was to use event-related potentials (ERPs) recording to understand the combination of automatic attentional orienting and the IOR effect. The ERP technique can provide more valuable information of the potential process in addition to the results of behavioral experiments. Moreover, as there is insufficient evidence about electrophysiological correlates of IOR and involuntary spatial orienting, the method can also investigate the consistency of behavioral results and ERP results. The current experiment sought to investigate the early stage of IOR with sensory/perception component in the visual spatial attention field. Electrophysiological components like P1, N1, Nd, and N2pc were used to investigate the modulations of peripheral cueing associated with behavioral facilitation or inhibition effects for different research purposes. An enhancement of the P1 component (larger amplitude for cued location trials than uncued ones) was usually associated with the facilitatory effect (Doallo et al., 2004), while a reduction of the P1 component was associated with the IOR effect (McDonald et al., 1999; Prime and Jolicoeur, 2009; Satel et al., 2013). Additionally, the behavioral IOR effect has been observed in the absence of the P1 effect (Wang et al., 2012). Therefore, researchers have concluded that P1 may not be a direct marker of IOR (Prime and Ward, 2006; Jones and Forster, 2012, 2014; Satel et al., 2014). The facilitatory effect associated with N1 may be enhanced for cued trials rather than uncued ones (Eimer, 1994; Martín-Arévalo et al., 2014), and the IOR effect had a reversal with enhanced amplitude of N1 for uncued rather than cued trials (Prime and Ward, 2004; Prime and Jolicoeur, 2009; Satel et al., 2014). However, there was still an inconsistent association between N1 and behavioral cueing effects (Hopfinger and Mangun, 1998; Prime and Ward, 2006; Chica and Lupiáñez, 2009). The Nd component can be observed in most experiments, but there was no agreed upon explanation of the underlying mechanism. Researchers found a potential association that may reflect the IOR effect of Nd at the occipital electrodes (Satel et al., 2013, 2014), which refers to a reduction of the Nd component at the cued location. The first N2pc study of IOR was investigated in the covert deployment of attention (McDonald et al., 2009). They found reduced amplitude at the cued location relative to the uncued location and explained that the inhibitory process underlying IOR reduced the probability of attention returning to cued location. Recently, researchers found a reduction of the N2pc component associated with spatial attention of the IOR effect on the cued trials (Martín-Arévalo et al., 2014). Having said that, further research should be conducted due to the lack of experimental data of N2pc. Since behavioral predictions and electrophysiological modulations related to facilitation and IOR effects depend on many variables, there was no single electrophysiological marker for facilitation and IOR (Martín-Arévalo et al., 2016).

Considering that IOR is independent of either voluntary or involuntary spatial orienting (Chica and Lupiáñez, 2009; Martín-Arévalo et al., 2013), we hypothesize that IOR will not be mediated by non-predictive central cues. Another crucial hypothesis is that exogenous (peripheral cue) and endogenous (central cue) orienting mechanisms may contribute independently to performance and each mechanism has its own representative and independent effect (Berger et al., 2005; Chica et al., 2013). In line with behavioral outcomes, there will also be a disassociation between IOR and spatial orienting at the neural level.

## Materials and Methods

“This study was approved by the Human Ethics Committee of Nantong University. A written informed consent was obtained from all the participants before their participation.”

### Participants

Twenty three paid university participants (13 female, 10 male; aged 19–23 years, mean age 21) volunteered to participate in the study. All participants had normal or corrected-to-normal vision, were right-handed, were without mental illness and color blindness, and hadn’t participated in a similar experiment before.

### Stimuli and Apparatus

All stimuli were drawn in white and presented on a black background. Each trial started with two rectangular boxes and a fixation. Each box was 2.34° (horizontal) × 3.36° (vertical) and fixation was 0.5° (horizontal) × 0.5° (vertical). The peripheral cue was a solid circle appearing centrally in one of two rectangular boxes. The central cue was represented by an arrow with an arrowhead and arrow-tail (pointing to the right or left) or a straight line (catch trials). The target was a hollow circle, the same size as the peripheral cue, and appearing in the right or left box. Participants sat in a dimly lit and electromagnetically shielded room, with their heads located 70 cm away from the computer screen. The experiment was presented with E-prime software, which controlled the presentation of the stimuli and the acquisition of data on a PC.

### Design and Procedure

The present experiment consisted of a 2 (peripheral cueing: cued vs. uncued) × 2 (central cueing: indicated vs. non-indicated) within-subject design. When the cue and target appeared at the same location, it was named cued trials. When the cue and target appeared at different locations, it was named uncued trials. An indicated or non-indicated trial referred to a target appearing at the location that was, or was not, indicated by the central cue. In the course of the experiment, participants were asked to locate the target, and their RTs, correct rate, and EEG data were recorded simultaneously.

**FIGURE 1:**
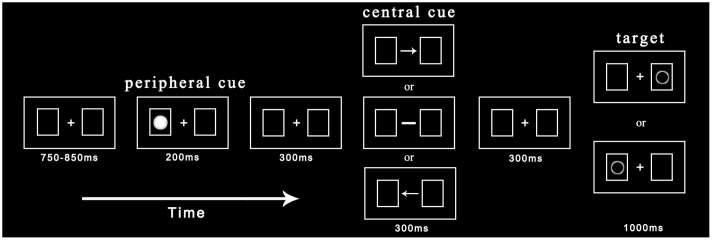
**Procedure of events in a sample trial.** The peripheral cue and target appear randomly at the left or right location with equal probability while the central cue points to the left or right. Under indicated and non-indicated conditions, cued and uncued locations have the same number of trials.

Before entering the laboratory, participants were explained the basic principles of ERP recording in order to eliminate their tension. They were then required to give written informed consent. The experimental procedure is depicted in **Figure 1**. Each trial began with the presentation of central fixation and two rectangular boxes with a variable duration between 750 and 850 ms. Participants needed to maintain their fixation on a cross during the experiment. In one of two possible boxes, a non-predictive cue (solid circle) was presented randomly with equal probability for 200 ms. After a 300 ms delay, the cross fixation was replaced by the central cue (arrow or straight line) for 300 ms. The central cue appeared with equal probability toward the left or right and was non-predictive for the target location. Subsequently, after another 300 ms delay, the target (hollow circle) appeared in the left or right box with equal probability until participants made a response or 1000 ms had elapsed. Participants were asked to judge the target location as soon as they found the target: if the target appeared in the left box, participants were instructed to press the “Z” key; if the target appeared in the right box, participants were instructed to press the “M” key. Each trial ended with a black blank screen for 2000 ms.

Prior to formal experimental blocks, each participant needed to complete practice trials. There were a total of 24 trials per practice block for each participant. The formal experiment would not start until the participants understood the experimental requirements. This would enable them to have a correct rate of at least 90%. The formal experiment contained four blocks of 416 trials. Each block had 104 trials, of which 8 were catch trials (under this condition, no target was presented and no response was needed). The remaining 96 trials consisted of 24 repetitions of the factorial combination of peripheral cueing and central cueing. Neither practice trials nor catch trials were analyzed. During the experiment, participants were told to try their best to reduce their actions of swallowing, frowning, blinking, and to keep their bodies from moving. Rest periods were provided at every block.

### ERP Recording and Analysis

Electroencephalogram (EEG) signals were recorded using a 64 Ag-AgCl electrodes elastic cap placed according to the international 10-20 system and the Neuroscan ERP workstation (scan4.5), with the reference electrode on the left mastoid (M1) and a ground electrode (GND) on the medial frontal aspect. A vertical electrooculogram (EOG) was recorded from above and below the participant’s left eye, and a Horizontal EOG was recorded from the outer canthi of both eyes. All electrodes impedance was kept below 5KΩ. The sampling rate and band pass were 1000 Hz/channel and 0.05∼100 Hz. Every participant washed his or her hair in the laboratory first, and recording started until the impedance was stable (below 5KΩ) with the conductive paste on the scalp.

Three participants were excluded because of excessive EEG artifacts (accepted less than 65% of the trials). The EEG data recorded in the experiment was used by scan software for offline analysis. We fused EEG data with behavioral data, and the average of M1 and M2 was converted to a new reference. Ocular artifacts (mean EOG voltage exceeding ± 100 μV) related to blinks and vertical eye movements were removed from the EEG, and trials of incorrect or no responses were also removed at EEG epoch (-100 ms-500 ms, appearance of target used as zero point). After baseline correction, trials with excessive artifacts (±80 μV standard) were rejected for further analysis (acceptance rate of each participant was more than 85% of trials). Finally, mean waveforms under all conditions performed a band-pass filter containing a high-pass filter of 0.1 Hz and a low-pass filter of 30 Hz (24 dB/octave) (Luck, 2014; Satel et al., 2013, 2014). According to the overall average map and previous research, the ERP components were divided by the time windows in which they occurred: P1, 80∼140 ms; N1, 140∼200 ms; and Nd, 200∼300 ms. We selected electrodes in the occipital areas for statistical analyses of P1, N1 (Po5, Po6, Po7, Po8, P5, P6, O1, O2) and Nd (Po7, Po8, Pz) (Prime and Ward, 2006; Prime and Jolicoeur, 2009; Satel et al., 2014). SPSS 16.0 for Windows was used for repeated measures analysis of variance of behavioral data and ERP data.

## Results

### Behavioral Results

According to the 2 × 2 experimental design, we conducted statistical analyses of the mean response times and correct rate for each condition. Trials with no response, incorrect response and extreme response (RTs below 200 ms and above 900 ms) were not included for RTs analysis (4.58% of trials).

On correct rate, a 2 (peripheral cueing: cued vs. uncued) × 2 (central cueing: indicated vs. non-indicated) repeated measures analysis of variance (ANOVA) revealed a significant main effect of peripheral cueing, *F*(1,22) = 11.41, *p* < 0.01, η^2^ = 0.34, suggesting that IOR was observed with a higher correct rate at the uncued location (99.5% ± 0.13) than the cued location (99.1% ± 0.16). Neither main effects of central cueing nor interaction between peripheral cueing and central cueing (all *p* > 0.05) were significant.

**FIGURE 2:**
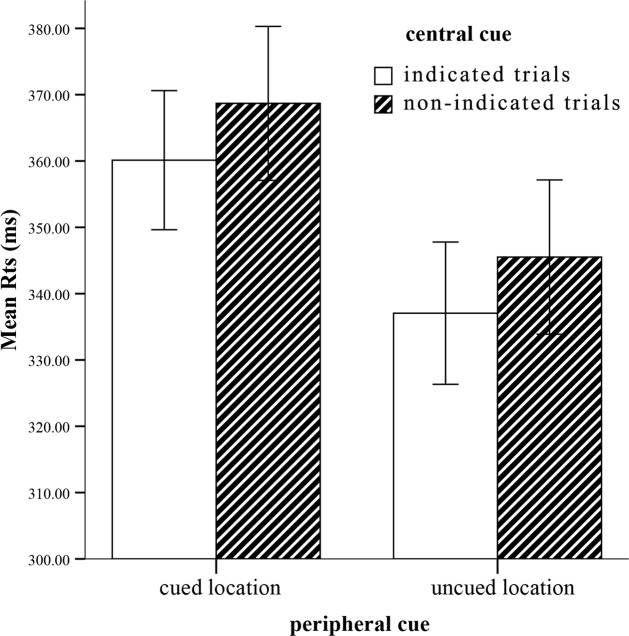
**Mean RTs for each condition.** Error bars show the SE of each condition.

On mean correct RTs (see **Figure 2**), a similar ANOVA revealed a strongly significant main effect of peripheral cueing, *F*(1,22) = 46.69, *p* < 0.0001, η^2^ = 0.68, indicating slower RTs at the cued location (364 ± 11) than the uncued location (341 ± 11). The central cueing effect was significant, *F*(1,22) = 5.95, *p* < 0.05, η^2^ = 0.21, reflecting faster RTs at the indicated location (349 ± 10) than the non-indicated location (357 ± 11). Crucially, interaction involving peripheral cueing and central cueing was not significant, *F*(1,22) = 0.001, *p* = 0.98, η^2^ = 0. The IOR effect sizes (cued RTs-uncued RTs) of the indicated cue (23 ms) and the non-indicated cue (23 ms) were not significant as well, *F*(1,22) = 0.001, *p* > 0.05. Thus, the peripheral cueing effect was independent of the central cueing effect.

### ERP Results

There was a three factors repeated measures ANOVA for mean amplitudes of the P1 and N1 components (see **Table 1**): peripheral cueing (cued vs. uncued), central cueing (indicated vs. non-indicated), and laterality (ipsilateral vs. contralateral).

**Table 1:** Mean ERP component (*M* ± *SE* μV) and peaks (ms) for each condition.

Cueing	P1		N1		Nd	
			
	Indicated	Non indicated	Indicated	Non indicated	Indicated	Non indicated
Cued	0.04 ± 0.26	0.06 ± 0.39	-1.4 ± 0.39	-1.12 ± 0.41	2.79 ± 0.46	2.9 ± 0.41
	116	115	172	170		
Uncued	0.21 ± 0.36	0.21 ± 0.31	-1.1 ± 0.43	-0.46 ± 0.33	3.97 ± 0.56	3.92 ± 0.53
	118	115	172	172		


P1: The peripheral cueing effect revealed no main effect of peripheral cueing, *F*(1,19) = 1.69, *p* = 0.21, η^2^ = 0.08, or central cueing, *F*(1,19) = 0.01, *p* = 0.91, η^2^ = 0.001, or laterality, *F*(1,19) = 1.68, *p* = 0.21, η^2^ = 0.08, and the interactions of peripheral cueing × central cueing, *F*(1,19) = 0, *p* = 0.98, η^2^ = 0, and of central cueing × laterality, *F*(1,19) = 1.65, *p* = 0.21, η^2^ = 0.08, were not significant. The interaction between peripheral cueing × laterality was marginally significant, *F*(1,19) = 3.84, *p* = 0.065, η^2^ = 0.17. The three-way interaction peripheral cueing × central cueing × laterality was also not significant, *F*(1,19) = 0.03, *p* = 0.88, η^2^ = 0.001.

N1: The analysis showed a significant main effect of peripheral cueing, *F*(1,19) = 4.84, *p* < 0.05, η^2^ = 0.2, with the amplitude of the uncued location (-0.86 μV) being smaller than the cued location (-1.14 μV). The main effect of central cueing was significant, *F*(1,19) = 4.72, *p* < 0.05, η^2^ = 0.2, with the amplitude of the non-indicated location (-0.79 μV) being smaller than the indicated location (-1.2 μV). The main effect of laterality was not significant, *F*(1,19) = 0.85, *p* = 0.37, η^2^ = 0.04. Importantly, both the interaction between peripheral cueing and central cueing, *F*(1,19) = 0, *p* = 0.99, η^2^ = 0, and the interaction between peripheral cueing and laterality, *F*(1,19) = 0.1, *p* = 0.76, η^2^ = 0.01, were not significant. The interactions of peripheral cueing × laterality, *F*(1,19) = 0.09, *p* = 0.77, η^2^ = 0.01, and of peripheral cueing × central cueing × laterality, *F*(1,19) = 0.83, *p* = 0.38, η^2^ = 0.04, were also not significant.

Mean amplitudes of the Nd components was assessed in a three-way, repeated-measures ANOVA with factors for peripheral cueing (cued, uncued), central cueing (indicated, non-indicated), and electrodes (Po7, Po8, Poz). The peripheral cueing effect was significant, *F*(1,19) = 26.47, *p* < 0.001, η^2^ = 0.58, with the amplitude of the uncued location (3.94 μV) being larger than the cued location (2.85 μV). The central cueing effect was not significant, *F*(1,19) = 0.05, *p* = 0.83, η^2^ = 0.002. Most importantly, the interaction between peripheral cueing and central cueing was not significant, *F*(1,19) = 0.17, *p* = 0.69, η^2^ = 0.009. The interaction of peripheral cueing × electrodes, *F*(2,38) = 6.79, *p* < 0.001, η^2^ = 0.26, was significant, showing larger cueing effects at the Poz electrodes. The interactions of central cueing × electrodes, *F*(2,38) = 2, *p* = 0.31, η^2^ = 0.06, and of peripheral cueing × central cueing × electrodes, *F*(2,38) = 0.14, *p* = 0.87, η^2^ = 0.01, were not significant.

**FIGURE 3:**
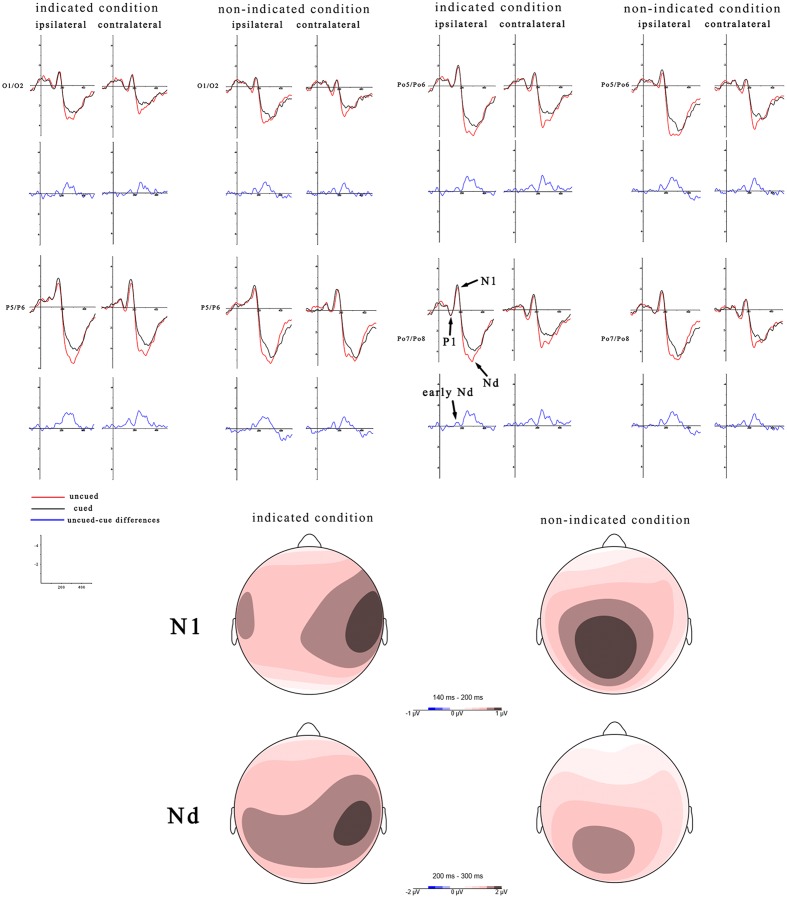
**Target-elicited ERP waveforms and topographic maps for indicated trials and non-indicated trials.** Mean amplitudes of the P1, N1, and Nd components are calculated during 80–140 ms, 140–200 ms, and 200–300 ms, with the red line indicating uncued trials and the black line indicating cued trials. Another early Nd was calculated during 100–200 ms. Red indicates the most positive activation in topographic heat maps. The left side shows ERPs over ipsilateral hemisphere and right are ERPs contralateral to target side. The blue lines and topographic maps reflect the cued-uncued differences. The scaling of topographic maps for the N1 is -1 to 1 μV and for the Nd is -2 to 2 μV.

According to the difference waves of **Figure 3**, mean amplitudes of an early Nd (during 100–200 ms latency window) were assessed in a three-way, repeated-measures ANOVA (peripheral cueing × central cueing × laterality). The results showed that, the peripheral cueing effect was significant, *F*(1,19) = 4.54, *p* < 0.05, η^2^ = 0.19, and the ERPs were more negative on cued trials (-0.44 μV) than uncued trials (-0.22 μV) at both contralateral and ipsilateral sites. The main effect of central cueing was significant, *F*(1,19) = 5.2, *p* < 0.05, η^2^ = 0.22, showing more negative amplitudes on indicated trials (-0.46 μV) than non-indicated trials (-0.21 μV). The main effect of laterality was not significant, *F*(1,19) = 0.41, *p* = 0.53, η^2^ = 0.02. The interactions of peripheral cueing × central cueing, *F*(1,19) = 0.26, *p* = 0.62, η^2^ = 0.01, of peripheral cueing × laterality, *F*(1,19) = 0.65, *p* = 0.43, η^2^ = 0.03, of central cueing × laterality, *F*(1,19) = 0.49, *p* = 0.5, η^2^ = 0.03, and of peripheral cueing × central cueing × laterality, *F*(1,19) = 0.83, *p* = 0.37, η^2^ = 0.04, were not significant.

## Discussion

In order to further explore the relationship between automatic attentional orienting operated by a non-informative central cue and the IOR effect operated by a peripheral cue, we used a location task to simultaneously examine these two mechanisms from the aspects of behavior and electrophysiology. Consistent with our hypothesis, the present results replicated the findings of previous studies, showing that IOR was independent of involuntary spatial orienting in both behavioral performance and neural correlates. There were three main results: First, we found a significant main effect of IOR on the behavior and Nd components. Second, a significant central cueing effect was observed on the behavior and N1 component. Third, the peripheral cueing effect and the central cueing effect did not interact crucially with each other on the behavioral and electrophysiological levels. This indicated that IOR and involuntary orienting of spatial attention might not be mutually interrelated mechanisms.

In the present experiment, the peripheral cue succeeded in eliciting the IOR effect. For central cue, recent studies have two different explanations as mentioned in the introduction section. Green et al. (2013) found the facilitation of central cue only at the long duration cues for the short SOA, and they explained this with cue-target conflict. Nevertheless, Gayzur et al. (2014) found the facilitation of gaze cue at both the short and the long duration for both the short and the long SOA, and they employed an explanation of reflexive orienting. The paradigm of present experiment is different with Green et al. (2013) and Gayzur et al. (2014), but it is similar to Martín-Arévalo et al. (2013). Due to the different paradigms among Green et al. (2013), Martín-Arévalo et al. (2013), and Gayzur et al. (2014), the central cueing effects of Martín-Arévalo et al. (2013) and the present experiment were not pure arrow cue effect. Long SOA was used in the present experiment and Martín-Arévalo et al. (2013), the results showed resembled facilitation of the arrow cue. Green et al. (2013) who explained the result as cue-target conflict for non-predictive cues, and this cue-target conflict only appeared in the long duration (cue and target stimuli overlapped temporally). The present experiment did not make central cues and the target present simultaneously, thus the differences between indicated and non-indicated trials were not suitable for the explanation of cue-target conflict. Green et al. (2013) also did not find any cueing effect of arrow cue at the short duration, but Gayzur et al. (2014) found cueing effect at the short duration for the short SOA. This may lead to their different explanations for the central cueing effect. Moreover, for the long duration condition of reflexive orienting, Martín-Arévalo et al. (2013) found the central cueing effect at the long SOA and Gayzur et al. (2014) found that at both the short SOA and long SOA. It seemed that the spatial-conflict was supported only at the short SOA for the long duration. For the short duration condition, the central cueing effect was not affected by the return cue condition and this facilitation of non-predictive cues was significant up to 1440 ms (McKee et al., 2007). Tipples (2002) used short duration with an interval of 25 or 225 ms between non-predictive central cue and target, and found faster RTs which indicated automatic orienting at the valid location than those at the invalid location. The same pattern of data was observed when the present experiment adopted an interval of 300 ms after central cue. Thus, the non-predictive arrow used in this atypical paradigm may also reflect the automatic orienting. In addition, dissociation of attention from the attended location is considered as a core factory for generating the IOR effect (Posner and Cohen, 1984; Klein, 2000). Regardless of indicated or non-indicated positions, mean RTs or correct rate of the cued location was slower or lower than the uncued location which referred to an IOR effect. In other words, IOR has been observed when attention was not disengaged from the attended location. These results were highly compatible with previous studies (Berlucchi et al., 2000; Berger et al., 2005; Chica et al., 2006; Chica and Lupiáñez, 2009; Martín-Arévalo et al., 2013). Therefore, the present behavioral result disproved the “reorienting hypothesis” of IOR.

The underlying neural process of cueing effects has been widely investigated by ERPs (McDonald et al., 2009; Satel et al., 2012, 2014). Researchers were interested in exploring which components can better represent electrophysiological markers of IOR and facilitation in visual attention (Lupiáñez et al., 2006; Martín-Arévalo et al., 2016). However, there still remained many debates or contradictory results (Prime and Ward, 2006; Prime and Jolicoeur, 2009). In the present experiment, we first chose three ERP components (P1, N1, and Nd) to explore insights into the relationship of IOR and the central cueing effect. The P1 amplitudes did not have any effect. The N1 amplitudes were enhanced for both cued and indicated trials, but not for uncued and non-indicated trials. These two results were very similar to the findings of Satel et al. (2014). In their experiment, behavioral IOR was observed, but P1 reduction was absent in the central arrow condition. They explained that P1 reduction would disappear when the cue and target were not peripheral onsets. According to previous studies, this enhancement of N1 used to be associated with the facilitatory effect (Eimer, 1994; Doallo et al., 2005; Martín-Arévalo et al., 2014). However, considering the current 8-ms difference between indicated and non-indicated trials during one SOA, this central cueing effect is not possible to unequivocally ascribe a small facilitatory effect to attention. Although the early N1 component seemed certainly plausible, it was not sure that this was the true N1 component because its time course was not close to the N1 peak. According to the difference waves, there seemed to be an early negative difference (Nd, subtracting invalidly cued amplitudes from validly cued amplitudes) component (McDonald et al., 1999) in the time range of 100-200ms, reflecting a single ERP component with a long duration. Thus, these effects of N1 component which appeared to start in the P1 interval needed to be further analyzed. The result of the early Nd was similar to McDonald et al. (1999) who investigated the same component (e.g., called posterior Nd in 120–200 ms) and found this difference began at the onset of the P1 and continued for nearly 100 ms after the N1 peak. Certainly, this early posterior negative difference (Nd) is unrelated to the N1. Two alternative interpretations will be discussed below. The early Nd effect spanning the P1 and N1 peaks (100–200 ms) was less positive on cued trials than that on uncued trials. This explanation was fitted well with perceptual inhibition which found smaller amplitudes of cued location for the early ERP components (Prime and Jolicoeur, 2009). On the other hand, this sustained Nd effect called reduced positivity that was also unrelated to the P1 amplitude. This different effect of peripheral cueing effect might indicate that it could arise from sensory refractoriness (McDonald et al., 1999).

Recently, Satel et al. (2014) found that Nd might be a more reliable neural marker of IOR. The Nd component reflecting a relation with IOR was often investigated at occipital electrodes during a time window ranging approximately from 200 to 300 ms (Prime and Ward, 2004, 2006; Wascher and Tipper, 2004; Satel et al., 2013). The Nd component had been related to the facilitatory effect for enhancement of the cued location (Eimer, 1993, 1994; Martín-Arévalo et al., 2014) and to the IOR effect for reduction of the cued location (Prime and Jolicoeur, 2009; Satel et al., 2012; Gutiérrez-Domínguez et al., 2014). The present experiment showed an inhibitory effect of Nd. Compared to the uncued location, the amplitude of Nd was smaller at the cued location in both indicated and non-indicated trials. Moreover, interaction effect between peripheral cue and central cue was not significant. This performance of the Nd component indicated that IOR existed at the later process stage, which may refer to the decision-making process (McDonald et al., 1999).

In summary, the current experiment found a highly coincident result showing dissociation between IOR and automatic spatial orienting in the behavioral data and the potential neural marker of Nd for IOR. It was generally proposed that IOR could be observed without disengagement from the attended location.

## Author Contributions

FP and XW designed and coordinated the study; XW and LZ carried out experiments and data processes; XW drafted the manuscript and FP reviewed the manuscript. All authors gave final approval for publication.

## Conflict of Interest Statement

The authors declare that the research was conducted in the absence of any commercial or financial relationships that could be construed as a potential conflict of interest.
